# Comparison of Flavonoid Content, Antioxidant Potential, Acetylcholinesterase Inhibition Activity and Volatile Components Based on HS-SPME-GC-MS of Different Parts from *Matteuccia struthiopteris* (L.) Todaro

**DOI:** 10.3390/molecules29051142

**Published:** 2024-03-04

**Authors:** Xin Wang, Jiatao Guo, Siqi Zang, Baodong Liu, Yuhuan Wu

**Affiliations:** 1 College of Life Science and Technology, Harbin Normal University, Harbin 150025, China; wangxin@hrbnu.edu.cn (X.W.); gjt05gjt03@126.com (J.G.); 99bd@163.com (B.L.); 2 College of Life and Environmental Sciences, Hangzhou Normal University, Hangzhou 310036, China

**Keywords:** *Matteuccia struthiopteris*, volatile components, HS-SPME-GC-MS, acetylcholinesterase inhibition activity, flavonoid content, antioxidant potential

## Abstract

*Matteuccia struthiopteris* is one of the most globally consumed edible ferns and widely used in folk medicine. Reports mainly focus on young fronds and the rhizome which are common edible medicinal parts. However, there are few detailed reports on other parts. Therefore, the volatile components of different parts based on HS-SPME-GC-MS were identified, and total flavonoid contents, antioxidant activities and acetylcholinesterase inhibitory activities were compared in order to reveal the difference of volatile components and potential medicinal value of different parts. The results showed that total flavonoid contents, antioxidant activities and volatile components of different parts were obviously different. The crozier exhibited the strongest antioxidant activities, but only underground parts exhibited a dose-dependent inhibition potential against AChE. Common volatile compounds were furfural and 2-furancarboxaldehyde, 5-methyl-. In addition, it was found that some volatile components from adventitious root, trophophyll, sporophyll and petiole were important ingredients in food, cosmetics, industrial manufacturing and pharmaceutical applications.

## 1. Introduction

Edible ferns have been an important part of vegetable consumption in many countries across the globe because of their high nutritional content [1]. *Matteuccia struthiopteris* (L.) Todaro is one of the most globally consumed edible ferns widely distributed in Asia, North America and Europe [2]. *M. struthiopteris* has a long history and is the homology of medicine and food, which was recorded as food in North America as early as 1939 and popular in China, Canada, Korea, United States, India, Russia, and Japan [3,4,5,6,7,8]. Young fronds, the rhizome, and sometimes the whole plant of *M. struthiopteris* are taken as food. Moreover, *M. struthiopteris* is widely used in folk medicine to treat several ailments; it is known for its antioxidant, anti-inflammatory, antibacterial, antiviral and antidiabetic activities and prevention of influenza [9]. The main compounds are starch, fatty acid [8,9], flavonoids, stilbenes, steroids and so on [10,11]. In addition, the essential oil from *M. struthiopteris* contains (*E*)-phytol, nonanal, and decanal [7], which shows considerable potential development prospect.

*M. struthiopteris* has been divided into six parts, including adventitious root, trophophyll, sporophyll, rhizome, crozier (young fronds) and petiole, throughout the history of its life. However, until now, there have been few detailed reports on volatile components and active utilization of the six parts which resulted in the limitation of utilization of *M. struthiopteris*.

Compared with the traditional GC (Gas Chromatography) method, headspace solid-phase micro-extraction gas chromatography–mass spectrometry (HS-SPME-GC-MS) is much more efficient and accurate and can isolate volatiles from plants without solvents [12,13]. Therefore, the volatile components of different parts from *M. struthiopteris* based on HS-SPME-GC-MS were compared, and antioxidant activities and acetylcholinesterase inhibitory activities were analyzed in detail in order to reveal the differences of volatile components and potential medicinal value of different parts of *M. struthiopteris*.

## 2. Results and Discussion

### 2.1. Total Flavonoid Content of Different Parts from M. struthiopteris

**Figure 1:**
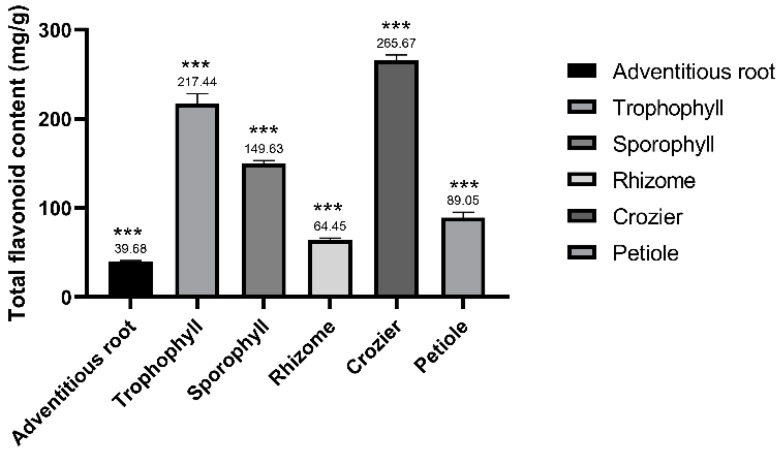
Total flavonoid content of different parts from *M. struthiopteris*. All data are presented as the mean ± SD. Error bars represent standard deviation and three replicates for each treatment. An unpaired two-tailed *t*-test was performed. *** *p* < 0.001.

Flavonoids are some of the vital function compounds in plant growth and stress resistance [14,15]. They shows antioxidant activities and health function [16,17]. In our previous reports, it was found that total flavonoid content of fern was much higher than that of bryophytes and some spermatophytes [18,19]. In this paper, total flavonoid contents of different parts from *M. struthiopteris* including the adventitious root, trophophyll, sporophyll, rhizome, crozier and petiole were obviously different (Figure 1). Thereinto, the total flavonoid content of crozier was the highest (265.67 ± 6.25 mg/g) and similar to that of trophophyll (217.44 ± 11.01), but the total flavonoid content of adventitious root was the lowest (39.68 ± 1.38 mg/g). An unpaired two-tailed t-test showed that the differences were extremely significant (*p* < 0.001) among different parts of *M. struthiopteris*. Significant differences might be closely related to the physiological functions of plant organs. Trophophyll of fern is an important organ for capturing the sun’s energy by photosynthesis, and crozier is the part with high plant auxin content. It was proven that flavonoids can stimulate the process of photosynthesis and synthesis of plant auxin [20], which might be result in the high total flavonoid contents of crozier and trophophyll.

### 2.2. Antioxidant Potential of M. struthiopteris

**Figure 2:**
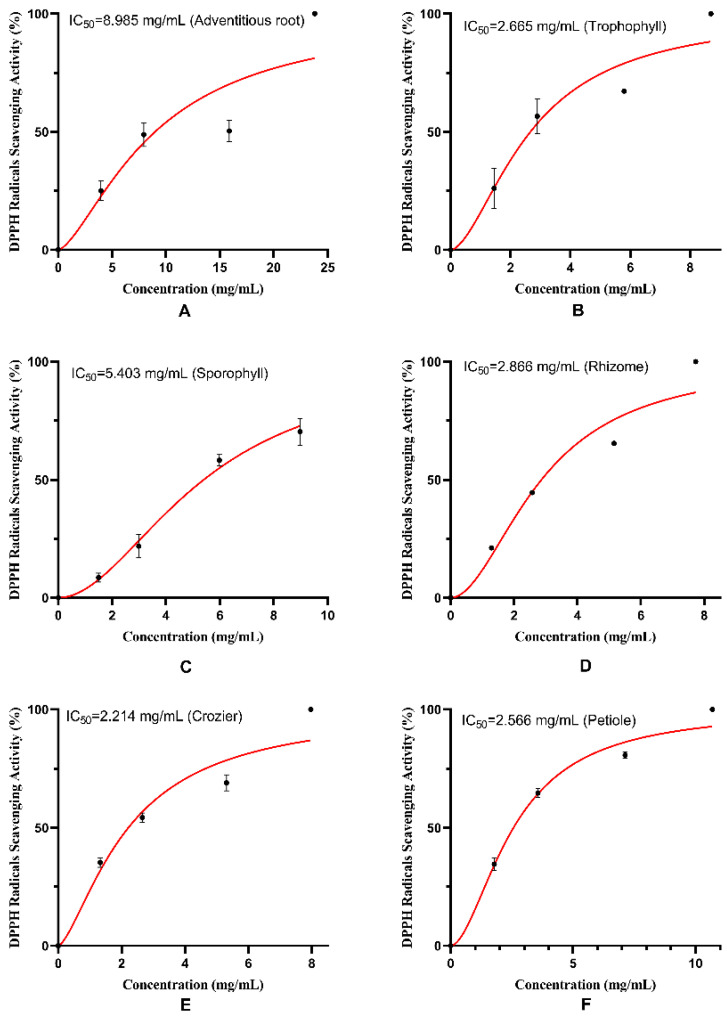
DPPH radical scavenging activities of the extracts from six parts of *M. struthiopteris:* (**A**) adventitious root, (**B**) trophophyll, (**C**) sporophyll, (**D**) rhizome, (**E**) crozier, (**F**) petiole. (red lines and black dots showsed the tendency of different concentrations of extracts to scavenge DPPH radicals).

**Figure 3:**
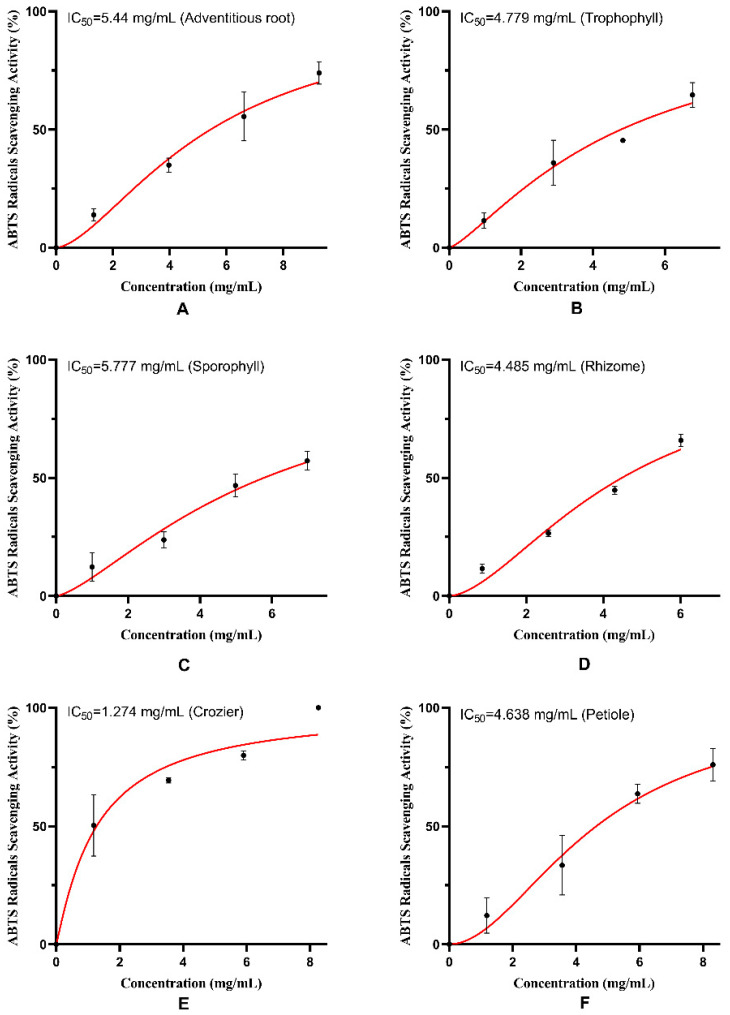
ABTS radical scavenging activities of the extracts from six parts of *M. struthiopteris:* (**A**) adventitious root, (**B**) trophophyll, (**C**) sporophyll, (**D**) rhizome, (**E**) crozier, (**F**) petiole. (red lines and black dots showsed the tendency of different concentrations of extracts to scavenge ABTS radicals).

DPPH free radical and ABTS free radical scavenging activities of the extracts from six parts of *M. struthiopteris* (adventitious root, trophophyll, sporophyll, rhizome, crozier and petiole) are shown in Figure 2 and Figure 3. It was found that six parts of *M. struthiopteris* all showed strong DPPH free radical and ABTS free radical scavenging activities, which confirmed strong antioxidant activities of the extract of fern again [18]. The extract of crozier showed the strongest antioxidant activities, but adventitious root and sporophyll exerted weak radical scavenging activity.

### 2.3. Acetylcholinesterase Inhibition Activity

**Figure 4:**
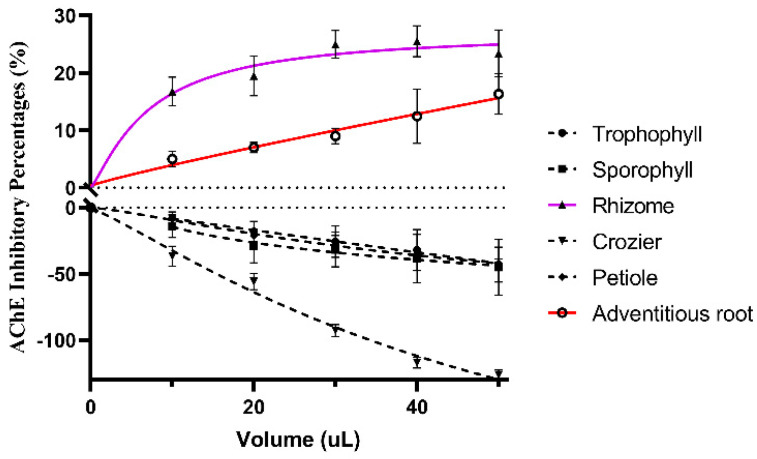
AChE inhibitory percentages (%) of the extracts from six parts of *M. struthiopteris* (adventitious root, trophophyll, sporophyll, rhizome, crozier and petiole).

AChE has proven to be the most viable therapeutic target for symptomatic improvement in AD (Alzheimer’s disease). So far, huperzine A has been a better potent acetylcholinesterase (AChE) inhibitor than tacfin [21]. However, the content of huperzine A in Huperzia serrata is too low, and the species of Huperzia serrata are limited [22]. The discovery of new natural active ingredients for inhibiting AChE is urgent. In this paper, acetylcholinesterase inhibition activity of different parts from M. struthiopteris are determined in Figure 4. The results showed that only the extracts of underground parts (adventitious root and rhizome) exhibited a dose-dependent inhibition potential against AChE.

### 2.4. Volatile Components of M. struthiopteris by HS-SPME-GC-MS

As vital and potential medicinal compounds, volatile components showed extensive economic value [23,24,25]. In 2007, Miyazawa et al. investigated the essential oil obtained from above-ground parts of *M. struthiopteris* with diethyl ether, which enriched the utilization of *M. struthiopteris*. In this paper, the volatile components of adventitious root, trophophyll, sporophyll, rhizome, crozier and petiole were all identified and comparatively analyzed for comprehensive and in-depth development.

#### 2.4.1. Volatile Components of *M. struthiopteris*

**Figure 5:**
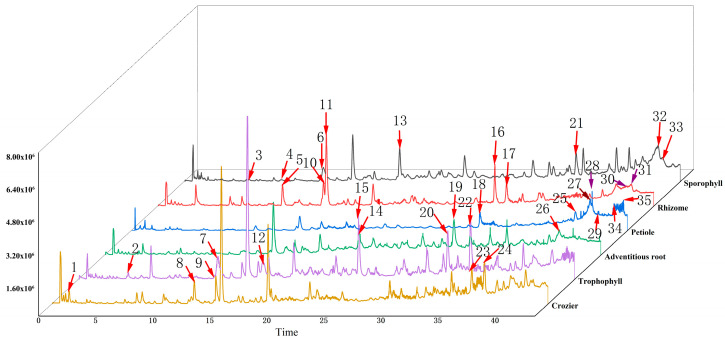
Volatile components with area percentage greater than 3% of six parts of *M. struthiopteris* (adventitious root, trophophyll, sporophyll, rhizome, crozier and petiole). The compounds (Numbers 1–35) were listed in Table 1.

A total of 306 volatile components were found in *M. struthiopteris* with HS-SPME GC-MS in this paper (Table S1). A total of 35 compounds (Figure 5) with area percentage greater than 3% are listed in Table 1, including hydrocarbon, aldehydes, alcohols, benzene, acids, ketenes and ethers.

#### 2.4.2. Comparative Analysis of Volatile Components from Different Parts of *M. struthiopteris*

**Figure 6:**
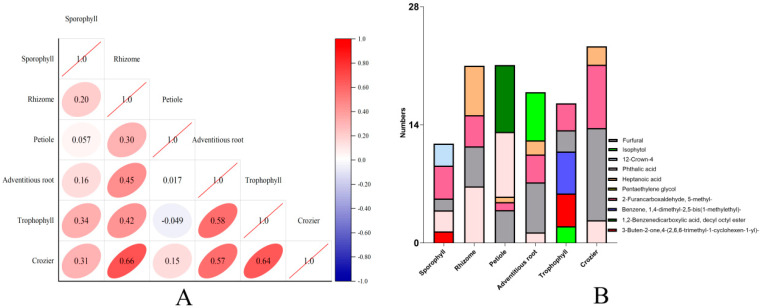
Comparative Analysis of volatile components of different parts of *M. struthiopteris.* (**A**): Correlation analysis of volatile components; (**B**): The distribution of main volatile components.

Conducting further analysis based on the results, it was found that the types and contents of volatile components of different parts from *M. struthiopteris* were significantly different, and strong positive correlations between young crozier and rhizome, adventitious root and trophophyll were detected (Figure 6A). The main volatile components type from young crozier and rhizome of *M. struthiopteris* is same, but the content is different, which might explain the strong correlations (Figure 6B). 

**Figure 7:**
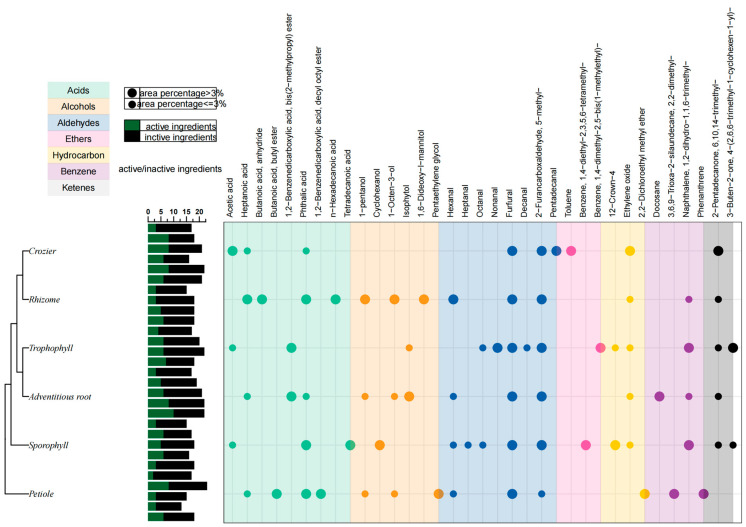
Distribution characteristics of volatile components from six parts of *M. struthiopteris* (adventitious root, trophophyll, sporophyll, rhizome, crozier and petiole).

Common compounds in adventitious root, trophophyll, sporophyll, rhizome, crozier and petiole of *M. struthiopteris* were furfural and 2-furancarboxaldehyde, 5-methyl- (Figure 6B and Figure 7). Among all the volatile components furfural was the volatile compound with the highest content of *M. struthiopteris*, mainly distributed in rhizome, trophophyll and crozier. 12-Crown-4 mainly distributed in sporophyll. The volatile compound with the highest content in petiole was phthalic acid.

The results showed that some volatile components of *M. struthiopteris* were important ingredients in food, cosmetics, industrial manufacturing and pharmaceutical applications. Furfural widely distributed in fruits was one of the most important aromatic compounds [26]. Meanwhile, furfural and its derivatives are widely applied in drugs, insecticides, food, even in industry [26,27]. It is worth noting that the content of furfural in crozier and trophophyll was the highest.

1-octen-3-ol was mainly distributed in rhizome of *M. struthiopteris* was an attractant for Anopheles and Aedes mosquitoes [28] and was able to inhibit *Monilinia fructicola* in vitro [29]. However, tetradecanoic acid was only distributed in sporophyll of *M. struthiopteris*; it showed larvicidal and repellent activity against *Aedes aegypti* (Linn.) and *Culex quinquefasciatus* (Say.) [30]. Isophytol mainly distributed in the adventitious root of *M. struthiopteris* is a fragrance ingredient used in the cosmetics industry as well as in non-cosmetic products such as household cleaners and detergents. Ethylene oxide found in the crozier of *M. struthiopteris* is commonly used in medical treatment and food processing as a sterilizing agent [31,32].

Phenanthrene (only distributed in petiole) and 1, 2-benzenedicarboxylic acid, bis (2-methyl propyl) ester (mainly distributed in the adventitious root) both showed biological toxicity [33]. Nonanal and decanal only found in the trophophyll of *M. struthiopteris* are antifungal constituents [34], and n-hexadecanoic acid only distributed in the rhizome of *M. struthiopteris* possesses antioxidant, antibacterial and anti-Inflammatory activities [35].

In addition, based on antioxidant potential and AChE inhibition of different parts of *M. struthiopteris*, it was speculated that furfural and ethylene oxide might be related to DPPH free radical and ABTS free radical scavenging activities, but isophytol and 1,2-benzenedicarboxylic acid, bis(2-methylpropyl) ester might be related to acetylcholinesterase inhibition activity.

## 3. Materials and Methods

### 3.1. Plant Materials

**Figure 8:**
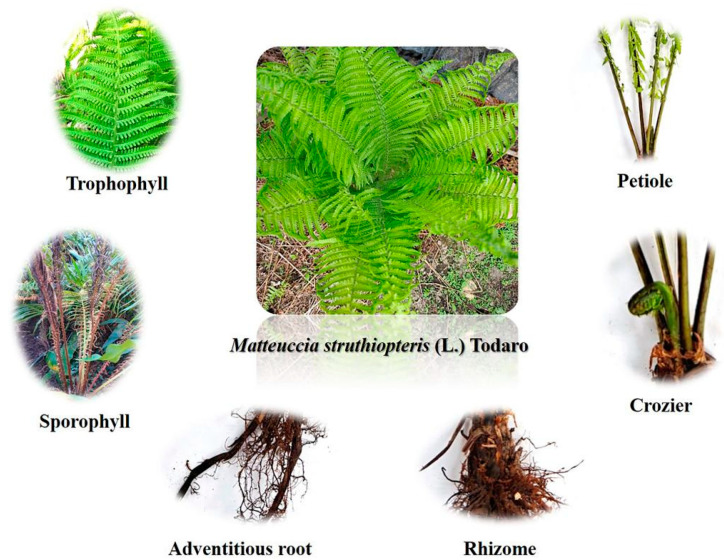
Different parts from *M. struthiopteris*.

*Matteuccia struthiopteris* (Figure 8) was collected on 29 September 2020 from the Botanical Garden of Harbin Normal University, where a greenhouse served for scientific research and teaching with proper temperature (18–35 °C), luminous intensity (1500 Lx–3000 Lx) and relative humidity (35~80%) identified by Prof. Baodong Liu.

### 3.2. Chemicals and Reagents

Rutin (purity > 99.0%), 2,2-diphenyl-1-picrylhydrazyl (DPPH), 2,2′-azinobis-(3-ethylbenzothiazoline-6-sulfonic acid) (ABTS), and 5,5′-dithiobis-(2-nitrobenzoic acid) (DTNB) were purchased from Aladdin Reagent Int. (Shanghai, China). Acetylcholinesterase was purchased from Yuanye Bio-Technology Co. (Shanghai, China). Acetylthiocholine (ATCh) was purchased from Macklin Bioc-Technology Co. (Shanghai, China).

### 3.3. Preparation of Plant Extracts

Fresh plants were collected and separated into six parts, including adventitious root, trophophyll, sporophyll, rhizome, crozier and petiole. One sample including six new-collected parts was cleaned quickly and stored in a Ziplock bag for HS-SPME-GC-MS analysis. The other sample was first kept in the shade and then dried at 75 °C in a drying oven for 12 h. The dried and crushed samples were weighed at 1.00 g respectively, then extracted with 70% ethanol twice. The extraction process was conducted in a 50 °C water bath for 2 h; ultrasound-assisted extraction lasted for 20 min. The filtered extracts were prepared for determination of total flavonoid content and bio activities.

### 3.4. Determination of Total Flavonoid Contents

The method of determination of total flavonoid content was the same as described in our previous report [36]. Rutin was chosen for producing the calibration curves. Different concentrations of rutin extracted with 70% ethanol were mixed with 5% NaNO_2_ (0.3 mL, 6 min), 5% Al (NO_3_)_3_ (0.3 mL, 6 min) and 4% NaOH (4.4 mL, 12 min) in turn, and then the optical density (OD) values of the mixtures at 510 nm were recorded. The optical density values were linearly fitted, and the fitting equation was obtained (y = A + Bx). The determination process of total flavonoid content of the six parts was similar to above steps, and the following formula was used:

Total flavonoid content (mg/g) = [(OD_1_ + OD_2_ + OD_3_)/3 − A]/B × 10/2 × volume/100 × 100%

### 3.5. Antioxidant Potential (DPPH and ABTS Free Radicals Scavenging Assay)

Radical scavenging activity was determined by detecting the degree of free radical reduction. The methods of DPPH and ABTS free radical scavenging assay were the same as in our previous report [18]. DPPH (0.1 mM) dissolved in 70% ethanol was mixed with extracts of different concentrations in the dark for 30 min, and then optical density values were recorded at 517 nm. The mixture of ABTS (7 mM) dissolved in ultrapure water and potassium persulfate (2.45 mM) was kept in the dark for 12 h before use in order to form the stable blue–green cationic radical ABTS^+^, namely the ABTS solution. The extracts of different concentrations were mixed with a stable ABTS solution, and the absorbance value was recorded at 734 nm. The experiments described above were both performed in triplicate, and 70% ethanol was taken as control.

### 3.6. Acetylcholinesterase Inhibition Activity

AChE inhibition activity of extracts of the six parts was measured by the method adopted by Xiao et al. [37]. Acetylcholinesterase catalyzes the formation of choline from acetylcholine and choline reacts with dithio-p-nitrobenzoic acid (DTNB) to form 5-mercapto-nitrobenzoic acid (TNB) which shows a maximum absorption peak at 412 nm.

Samples of different concentrations were added to an acetylcholinesterase solution dissolved in a PBS buffer (PH = 8.0). After the mixture was mixed thoroughly, acetylcholine was added. The reaction was carried out in a 37 °C water bath for 25 min. After the reaction, DTNB was added to the solution for 5 min, and then optical density was recorded at 412 nm. The experiments described above were both performed in triplicate, and 70% ethanol was taken as control.

### 3.7. Volatile Component Analysis by HS-SPME-GC-MS

Instrumental parameters of volatile component analysis by HS-SPME-GC-MS were same with parameters in previous report [13].

### 3.8. Data Analysis

Statistical analysis was undertaken using the R software 4.3.1, Prism 8.0.1 and Origin 7.5. Data are reported as the mean of three independent samples.

## 4. Conclusions

Total flavonoid contents, antioxidant activities and volatile components of different parts from *M. struthiopteris* were obviously different, which showed that the exploitable values of different parts of *M. struthiopteris* were different. Specifically, the volatile components of trophophyll, sporophyll, petiole and the underground parts of *M. struthiopteris* were worthy of further development. Meanwhile, it was proven that the underground parts of *M. struthiopteris* with stronger antioxidant potential and AChE inhibition activity could probably be used for medicinal purposes. In addition, the microwave method and the enzymatic method could be used for isolating more bioactive molecules.

## Figures and Tables

**Table 1:** Compounds of *M. struthiopteris* with area percentage greater than 3%.

No.	Time	Compound	Type	Compound Formula	Sporophyll	Rhizome	Petiole	Adventitious Root	Trophophyll	Crozier
1	0.797	Ethylene oxide	Ethers	C_2_H_4_O	2.479	2.884	-	2.201	1.063	4.022
2	3.354	Hexanal	Aldehydes	C_6_H_12_O	0.12	3.003	0.619	0.322	-	-
3	5.516	Heptanal	Aldehydes	C_7_HO	0.207	-	-	-	-	-
4	8.5	Octanal	Aldehydes	C_8_H_16_O	0.24	-	-	-	0.354	-
5	10.9	1-pentanol	Alcohols	C_5_H_12_O	-	3.298	1.44	0.159	-	-
6	12.216	Cyclohexanol	Alcohols	C_6_H_12_O	3.785	-	-	-	-	-
7	12.249	Nonanal	Aldehydes	C_9_H_18_O	-	-	-	-	4.058	-
8	12.573	Toluene	Benzene	C_7_H_8_	-	-	-	-	3.081	3.081
9	14.4	Acetic acid	Acids	CH_3_COOH	0.517	-	-	-	1.327	3.121
10	14.5	1-Octen-3-ol	Alcohols	C_8_H_16_O	-	3.936	0.862	0.496	-	-
11	14.8	Furfural	Aldehydes	C_5_H_4_O_2_	5.266	11.442	3.883	7.175	13.369	13.621
12	16.178	Decanal	Aldehydes	C_10_H_20_O	-	-	-	-	2.591	-
13	18.9	2-Furancarboxaldehyde, 5-methyl-	Aldehydes	C_6_H_6_O_2_	3.88	3.691	0.954	3.305	3.182	7.493
14	22.33	Docosane	Hydrocarbon	C_22_H_46_	-	-	-	4.638	-	-
15	24.597	Naphthalene, 1,2-dihydro-1,1,6-trimethyl-	Hydrocarbon	C_13_H_16_	3.491	0.404	-	1.179	4.884	-
16	29.56	Heptanoic acid	Acids	C_7_H_14_O_2_	-	5.857	0.644	1.664	-	2.179
17	30.588	Butanoic acid, anhydride	Acids	C_8_H_14_O_3_	-	3.507	-	-	-	-
18	30.602	Butanoic acid, butyl ester	Acids	C_8_H_16_O_2_	-	-	3.888	-	-	-
19	30.607	Isophytol	Alcohols	C_20_H_40_O	-	-	-	5.729	1.969	-
20	32.38	3-Buten-2-one, 4-(2,6,6-trimethyl-1-cyclohexen-1-yl)-	Ketenes	C_13_H_20_O	1.37	-	-	-	3.88	-
21	34.374	Benzene, 1,4-diethyl-2,3,5,6-tetramethyl-	Benzene	C_14_H_22_	3.002	-	-	-	-	-
22	34.393	Benzene, 1,4-dimethyl-2,5-bis(1-methylethyl)-	Benzene	C_14_H_22_	-	-	-	-	4.986	-
23	36.812	Pentadecanal	Aldehydes	C_15_H_30_O	-	-	-	-	-	4.721
24	37.9	2-Pentadecanone, 6,10,14-trimethyl-	Ketenes	C_18_H_36_O	2.592	0.829	-	0.813	0.504	3.769
25	38.945	Phenanthrene	Hydrocarbon	C_14_H_10_	-	-	3.321	-	-	-
26	39.835	1,2-Benzenedicarboxylic acid, bis(2-methylpropyl) ester	Acids	C_16_H_22_O_4_	-	-	-	4.251	3.136	-
27	40.2	Phthalic acid	Acids	C_8_H_6_O_4_	3.853	6.695	13.15	1.245	-	2.679
28	40.412	1,2-Benzenedicarboxylic acid, decyl octyl ester	Acids	C_26_H_42_O_4_	-	-	7.934	-	-	-
29	40.983	2,2-Dichloroethyl methyl ether	Ethers	C_3_H_6_Cl_2_O	-	-	3.025	-	-	-
30	41.126	n-Hexadecanoic acid	Acids	C_16_H_32_O_2_	-	4.162	-	-	-	-
31	41.493	1,6-Dideoxy-l-mannitol	Alcohols	C_6_H_14_O_4_	-	3.753	-	-	-	-
32	41.545	12-Crown-4	Ethers	C_8_H_16_O_4_	11.753	-	-	-	1.904	-
33	41.936	Tetradecanoic acid	Acids	C_14_H_28_O_2_	3.241	-	-	-	-	-
34	42.331	Pentaethylene glycol	Alcohols	C_10_H_22_O_6_	-	-	5.139	-	-	-
35	43.093	3,6,9-Trioxa-2-silaundecane, 2,2-dimethyl-	Hydrocarbon	C_9_H_22_O_3_Si	-	-	3.871	-	-	-

## Data Availability

Data are available on public.

## Supplementary Materials

The following supporting information can be downloaded at: https://www.mdpi.com/article/10.3390/molecules29051142/s1, Table S1: *Matteuccia struthiopteris* compounds.
