# Supplementary material for: Comparison of Flavonoid Content, Antioxidant Potential, Acetylcholinesterase Inhibition Activity and Volatile Components Based on HS-SPME-GC-MS of Different Parts from Matteuccia struthiopteris (L.) Todaro
Source: Molecules. 2024 Mar 4;29(5):1142. doi: 10.3390/molecules29051142 (PMC10934419; doi:10.3390/molecules29051142)
Supplement: Supplementary file 1 [file molecules-29-01142-s001.zip › molecules-2874325-supplementary.pdf]

Time	Compound	Sporophyll	Rhizome	Petiole	Adventitious root	Trophophyll	Crozier
0.345	Acetamide			0.959			
0.511	L-Alanine, methyl ester			0.153			
0.797	Ethylene oxide	2.479	2.884		2.201	1.063	4.022
0.806	1,2-Propanediamine			0.939			
0.94	2-Pentanamine				0.227		
0.949	2-Ethylacridine			0.163			
0.959	2-Propanamine		0.345				
1.03	Ethanethiol					0.252	
1.097	Thieno[2,3-b]pyridine-2-carboxamide, 3-amino-6-methyl-			0.308			
1.204	Cyclotrisiloxane, hexamethyl-		0.611		0.479		
1.206	Acetone	0.583				0.173	
1.211	1H-Indole, 6-methyl-2-phenyl-			0.874			
1.214	Butane						0.381
1.383	Aminomethanesulfonic acid	0.265					
1.5	1-Propanol	0.642	0.667		0.36	0.069	
1.802	Benzene	0.21					
1.83	Cyclotetrasiloxane, octamethyl-		0.275	0.316	0.244		
2.097	2-Pentanone		0.222		0.301		
2.106	Butanal, 3-methyl-	0.212					
2.735	1,3,5-Cycloheptatriene	0.139					
2.792	2-Butenal				0.179		
3.001	Cyclohexene, 1-methyl-3-(1-methylethenyl)-,				0.323		
3.021	1,3-Propanediamine	0.347					
3.049	Carbamic chloride, diethyl-		0.139				
3.054	2-Butanone, 4-hydroxy-	0.121					0.38
3.073	2,4-Pentanedione						
3.354	Hexanal	0.12	3.003	0.619	0.322		
4.049	Cyclopentasiloxane, decamethyl-			0.225			
4.311	2-Propanol, 1-methoxy-	0.223			0.227	0.567	
4.854	Bicyclo[3.1.0]hexane, 4-methyl-1-(1-methylethyl)-, didehydro deriv.				0.135		
5.516	Heptanal	0.207					
5.606	Cyclohexene, 1-methyl-4-(1-methylethenyl)-, (.+/-.)-				0.662		0.918
5.721	1-Buten-3-yne	0.144					
6.359	Oxirane, ethenyl-	0.122					
6.368	2-Hexenal		1.184	0.238	0.478	1.744	
6.654	Furan	0.174	0.282		0.835		0.796
7.197	1,3,6-Octatriene, 3,7-dimethyl-				0.221		
7.4	3-Octanone		1.256				
7.9	3-Aminopyridine	0.39	0.856		0.479		0.952
8.487	Tetradecane				0.253		
8.5	Octanal	0.24				0.354	
8.887	1,2-Butadiene, 3-methyl-					0.335	
8.897	Phenylethanolamine	0.165					
8.94	1-Butanol, 2-ethyl-		0.302				0.838
9.021	Benzene, butyl-				0.343		3.769
9.7	Pyrazine	0.407	0.539		0.191		1.285
9.778	Benzene, 1,2,4-trimethyl-					0.414	
10.011	Pyrazine, 2,3-dimethyl-						0.644

10.068	2-Pyridinamine, 3-methyl-		0.424				1.75
10.9	1-pentanol		3.298	1.44	0.159		
11.973	Tridecane				0.346		
11.997	Pyrazine, 2-ethyl-5-methyl-						
12.035	3-Hexen-1-ol		0.463				
12.04	2,4-Hexadiene, (Z,Z)-			0.135			0.684
12.216	Cyclohexanol	3.785					
12.23	Pyrazine, 2-ethyl-3-methyl-						0.344
12.249	Nonanal					4.058	
12.421	2-Pentene, 3,4-dimethyl-, (E)-		1.359				0.916
12.497	3-Octanol				0.515		1.031
12.573	Toluene						3.081
13.349	2-Octenal, (E)-		0.351				
13.41	Decane				0.71	0.591	
13.411	Octane		0.464				
13.678	Oxalic acid, allyl undecyl ester	0.103					
13.683	Heptane, 4-ethyl-				0.186		
14.4	Acetic acid	0.517				1.327	
14.5	1-Octen-3-ol		3.936	0.862	0.496		
14.8	Furfural	5.266	11.442	3.883	7.175	13.369	13.621
15.788	2,4-Heptadienal, (E,E)-					1.239	4.022
15.8	Undecane	0.571	0.377				
15.85	Pentanal, 2-methyl-			0.81	0.753		
15.95	Pentasiloxane, dodecamethyl-		0.144		1.039		
16.178	Decanal					2.591	
16.3	Ethanone, 1-(1H-pyrazol-4-yl)-	0.672	1.003		0.447	1.241	
16.397	Pilocarpine			0.166			
16.683	Benzaldehyde	1.08	1.101	0.955	0.955	1.606	
16.688	Ethanone, 2-hydroxy-1-phenyl-			0.862			2.179
17.326	2-Cyclohexen-1-ol	0.248	1.16	0.158	0.83		2.679
17.5	Trichloroacetic acid, dodecyl ester	0.188		0.116	0.682		13.621
17.764	2-Tetradecene, (E)-				0.517		
17.84	Nonadecane	0.168		0.182			
17.878	2-Furanmethanol, acetate						
18.359	1,6-Octadien-3-ol, 3,7-dimethyl-						0.43
18.9	2-Furancarboxaldehyde, 5-methyl-	3.88	3.691	0.954	3.305	3.182	7.493
19.15	1H-Imidazole, 1,2-dimethyl-	0.778	0.763			1.04	
19.235	Triacontane						
19.416	Bicyclo[2.2.1]hept-2-en-7-ol				0.398		
19.6	Hexadecane	1.196	0.601	0.366	1.159	0.891	
20.135	1-Octadecanesulphonyl chloride				0.292		
20.326	Benzaldehyde, 4-methyl-					2.042	
20.345	2(3H)-Furanone, dihydro-4,4-dimethyl-		0.407				
20.407	Hentriacontane			0.218			
20.43	Heptacosane		1.011				
20.44	Nonacosane				1.095		
20.773	Naphthalene	0.544					
20.778	Heptadecane, 3-methyl-				0.626		1.046
20.797	Butyrolactone		0.454				
20.807	1,2,3-Trimethylindene					0.763	

20.873	Oxalic acid, allyl decyl ester						0.409
21.068	Heneicosane				0.665		
21.069	Trisiloxane, octamethyl-					0.28	0.618
21.075	Octacosane	0.485	0.159				
21.388	Trichloroacetic acid, pentadecyl ester					1.467	
21.459	Dichloroacetic acid, 3-pentadecyl ester				2.163		0.474
21.483	Acetophenone	1.722					
21.521	Sulfurous acid, octadecyl pentyl ester			0.25			
21.607	Benzeneacetaldehyde		1.473				1.414
21.616	Sulfurous acid, pentadecyl pentyl ester			0.246			
22.212	Decane, 2-methyl-					0.724	
22.254	2-Octenal, 2-butyl-			1.396			
22.33	Docosane				4.638		
22.335	Benzoic acid, 2,3-bis[(trimethylsilyl)oxy]-, trimeth	1.317	1.59				
22.35	Silane					0.482	
22.526	8-Heptadecene			0.298	0.597		
22.6	2-Furanmethanol	1.398	0.991			1.942	
23.188	2-undecanone	1.04					
23.192	1-Heptadecene				0.648		
23.254	Dodecane, 4-methyl-					0.79	
23.259	tricosane						
23.326	(2E)-Dodec-2-en-1-yl methyl ether	1.022					
23.364	2-Propen-1-amine, N-2-propenyl-		1.558				
23.378	Cyclotetradecane			1.087			
23.492	Benzene, 1,2,3,4-tetramethyl-					0.685	
23.569	Borneol				2.976		0.417
24.035	Octadecanol			0.157			
24.045	Cyclohexadecane	1.074					
24.064	Heptafluorobutyric acid, n-octadecyl ester					0.874	
24.069	1-Butyne, 3-methyl-		0.58		0.972		0.19
24.069	11,13-Dimethyl-12-tetradecen-1-ol acetate						
24.159	p-Menth-8(10)-en-9-ol, cis-				0.823		1.111
24.188	Behenic alcohol		0.187				
24.264	Bromoacetic acid, octadecyl ester		0.186				
24.302	Isophthalaldehyde					0.596	
24.597	Naphthalene, 1,2-dihydro-1,1,6-trimethyl-	3.491	0.404		1.179	4.884	
24.735	Nonahexacontanoic acid				0.944		
25.402	4-Methyl-E-9-octadecene		0.549				
25.411	1-Pentadecene				1.956		
25.421	7-Tetradecene, (E)-					0.812	0.437
25.435	1-Tridecene	1.452					
25.459	Trichloroacetic acid						
26.078	Ethanone, 1-(3-methylphenyl)-					0.398	
26.083	Trichloroacetic acid, hexadecyl ester				1.58		0.488
26.092	2-Hexen-4-yn-1-ol, (E)-		0.865				0.482
26.131	Benzoyl chloride, 3-methyl-/Ethanone	1.272					
26.2	Hexadecane, 2,6,10,14-tetramethyl-			1.144	1.932		
27.092	Phenol, 2-(1-methylethyl)-, methylcarbamate	0.325					
27.331	1-Nonadecene			0.165			2.294
27.4	Octadecane	0.375			1.006		

27.9	2,4-Decadienal	0.411	1.318	0.647	2.637		
28.3	E-14-Hexadecenal	0.355			0.998		
28.31	Pentafluoropropionic acid, hexadecyl ester		0.692			0.692	
28.554	1-(4-tert-Butylphenyl)propan-2-one					1.839	
28.559	Benzene, 1-(3-chloro-2-propenyl)-4-methoxy-	0.473					
28.65	Eicosane, 2-methyl-		0.18				
28.697	1H-Pyrrole, 1-(2-furanylmethyl)-	0.326					0.619
28.8	Cyclohexasiloxane, dodecamethyl-	0.328	0.344	0.397		0.297	
28.84	Fumaric acid, 3-heptyl tridecyl ester				1.413		
29.364	1,3-Cyclohexadiene, 1-methyl-4-(1-methylethyl)-					0.294	0.573
29.56	Heptanoic acid		5.857	0.644	1.664		2.179
29.816	Octadecyl trifluoroacetate				0.995		
29.821	5,9-Undecadien-2-one, 6,10-dimethyl-	1.193				0.573	
29.969	2,6-Octadien-1-ol, 2,7-dimethyl-						
30.145	1-(3-Hydroxyphenyl)urea						
30.164	2,3-Pyridinediamine					0.219	
30.588	Butanoic acid, anhydride		3.507				
30.593	Butanoic acid, 2-propenyl ester	2.875					
30.602	Butanoic acid, butyl ester			3.888			
30.607	isophytol				5.729	1.969	
30.631	Butanoic acid, 2-butoxy-1-methyl-2-oxoethyl ester						
30.826	Cyclohexene, 4-isopropenyl-1-methoxymethoxymethyl-			0.789			
30.869	Cyclohexanol, 2-methyl-5-(1-methylethyl)-		0.323				
31.012	2-Dodecen-1-yl(-)succinic anhydride			0.932			3.121
31.026	1-Ethynyl-3,trans(1,1-dimethylethyl)-4,cis-methoxycyclohexan-1-		0.263				
31.221	1-Decanol, 2-hexyl-			0.397			
31.307	Dotriacontyl heptafluorobutyrate			0.328			
31.373	2-Hexadecanol		0.317				
31.393	2-Piperidinone, N-[4-bromo-n-butyl]-			0.299			
31.478	17-Pentatriacontene			0.221			
31.526	Cyclopentane, 1,1,3-trimethyl-			0.157			
31.716	Phenylethyl alcohol		0.682				
31.731	Butylated Hydroxytoluene	0.15		1.678	0.349	0.34	
32	Tetradecanal	1.794	1.243		2.133	1.777	
32.35	3,5-di-tert-Butyl-4-hydroxybenzaldehyde				0.638		
32.38	3-Buten-2-one, 4-(2,6,6-trimethyl-1-cyclohexen-1-	1.37				3.88	
32.4	2-Nonadecanol		0.168	0.366			
32.721	Tetradecane, 1-chloro-					0.462	2.422
32.769	1-Tetradecene				0.227		
32.797	7-Tetradecene, (Z)-						
32.95	6,11-Undecadiene, 1-acetoxy-3,7-dimethyl-	0.49			0.552		
33.169	1,5-Anhydro-d-mannitol						0.988
33.264	1,6,6-Trimethyl-7-(3-oxobut-1-enyl)-3,8-dioxatricyclo[5.1.0.0(2,4)]octan-5-one					2.13	
33.4	Maltol		1.23				
33.526	Ethanone, 1-(1H-pyrrol-2-yl)-	1.069	0.825		0.426		
33.535	2-Hexenoic acid, (E)-					0.785	
33.54	Triethylene glycol			0.499			2.48
33.764	18-Crown-6, [2-(diethylboryl)phenyl]-			0.831			0.598
33.769	Chloroacetic acid, 3-pentadecyl ester		1.43				
33.78	Bromoacetic acid, hexadecyl ester	1.373			2.044		1.545

33.8	Bicyclo[3.1.1]heptane, 2,6,6-trimethyl-					0.767	
34.083	Cyclohexene, 6-(2-butenyl)-1,5,5-trimethyl-, (E)-	0.249					
34.174	2-Tridecanol			0.305			
34.197	1-(Methoxymethyl)-1H-benzotriazole				0.314		
34.374	Benzene, 1,4-diethyl-2,3,5,6-tetramethyl-	3.002					
34.393	Benzene, 1,4-dimethyl-2,5-bis(1-methylethyl)-					4.986	
34.435	Benzene, 1,3,5-trimethyl-				0.812		
34.559	2-Aminopyridine						
34.597	1-Methoxy-3-(2-hydroxyethyl)nonane			0.193			
34.997	1H-Pyrrole-2-carboxaldehyde	2.834	0.39		0.465		
35.026	2-Pentadecanone					0.485	
35.207	2-Heptanol					0.337	
35.22	Tridecanal		0.579	0.741			0.784
35.3	Hexadecanal				2.451		
35.621	Heptadecanol				0.492		
35.626	2H-1-Benzopyran-2-one, 4-methyl-						
35.654	1,4,7,10,13,16-Hexaoxacyclooctadecane		1.041	0.197		0.915	
35.678	1,7-Dihydroxynaphthalene	0.437					
35.731	2,3-Naphthalenediol						
35.869	Carbamic acid		0.515				
36	2-Chloroethyl methyl ether					0.258	1.025
36.117	Ethane, 1,1-diethoxy-		0.304				
36.221	Hexanoic acid					0.644	
36.235	1-Propanol, 2-(1-methylethoxy)-	0.239					
36.269	Carbamic acid, 1-methylethyl ester			0.363			
36.3	[2-[2-[2-[2-[2-[2-[2-[2-(tert-Butyldimethylsilyloxy)ethoxy]ethoxy]		0.981				
36.483	1-Chloro-4-(1-ethoxyethoxy)-2-methylbut-2-ene		0.321				
36.574	1,1-Diethyl-4-phenylsemicarbazide			1.058			
36.645	Thiophene, 2-propyl-				2.468		0.488
36.669	1-Nonene, 2-ethyl-3-(methoxymethoxy)	0.299					1.525
36.755	2-Furancarboxaldehyde, 5-(hydroxymethyl)-					2.035	0.757
36.759	2-Pentadecanol		2.417				
36.812	Pentadecanal						4.721
37.016	Ethane, 1-isothiocyanato-2-methoxy-			0.152			1.267
37.259	Heptaethylene glycol						
37.355	15-Crown-5						
37.392	2-[2-[2-[2-[2-[2-[2-(2-Hydroxyethoxy)ethoxy]ethoxy]ethoxy]ethox		1.674		0.608		
37.597	1-Methoxy-5-trifluoroacetoxyhexane		0.617				
37.621	Ethanol, 2-[2-(2-methoxyethoxy)ethoxy]-						
37.712	1,2-Cyclopentanedione, 3-(2-methoxyethyl)-			0.288			
37.893	Propanoic acid, 2-methyl-, methyl ester			0.717			
37.9	2-Pentadecanone, 6,10,14-trimethyl-	2.592	0.829		0.813	0.504	3.769
38.2	Hydrazine, (2-phenylethyl)-	0.637		0.599			
38.321	Benzenemethanamine, 3-fluoro-					0.625	1.053
38.331	Ethane, 1-bromo-2-methoxy-			0.933			
38.345	Dicyclohexano-24-crown-8				0.196		
38.54	Quinoline, 7-methoxy-	0.645					
38.564	2,5,8,11,14-Pentaoxahexadecan-16-ol			1.121			
38.631	Ethanol, 2-(vinyloxy)-				0.16		
38.945	Phenanthrene			3.321			

38.969	Propanoic acid, 2-hydroxy-, butyl ester				1.514		
39.002	2-Heptanol, 6-methyl-		1.269				
39.012	Nonanoic acid	2.518				1.711	
39.255	4-Penten-2-ol					0.378	
39.26	2-Tetradecanol	0.612	0.434		0.617		1
39.421	4-Methoxy-2-methylbut-1-ene			2.275			
39.6	Ethanone, 1-(2-hydroxy-5-methylphenyl)-	1.522					
39.626	3,6,9,12,15-Pentaoxanonadecan-1-ol					0.466	
39.835	1,2-Benzenedicarboxylic acid, bis(2-methylpropyl) ester				4.251	3.136	
39.931	3,5-Bis(methoxymethoxy)-4-nitrotetrahydrothiopyran						
40.2	Phthalic acid	3.853	6.695	13.15	1.245		2.679
40.412	1,2-Benzenedicarboxylic acid, decyl octyl ester			7.934			
40.445	Toluene, 2-[(2,3-dimethoxy)propoxy]-				1.1		
40.45	Phthalic acid, cylclohexylmethyl pentyl ester						
40.778	1,3-Dioxolane, 2-(dichloromethyl)-				1.23		
40.983	2,2-Dichloroethyl methyl ether			3.025			
41.059	(Methoxymethyl)trimethylsilane				1.315		
41.126	n-Hexadecanoic acid		4.162				
41.2	Methoxyacetic acid, hexyl ester				1.115		
41.283	2-Butanol, 3-methyl-			1.838			
41.459	Formic acid, 1-methylpropyl ester			2.634			0.275
41.493	1,6-Dideoxy-l-mannitol		3.753				
41.54	Butanamide, 3,N-dihydroxy-						
41.545	12-Crown-4	11.753				1.904	
41.588	Propanoic acid, 3-methoxy-, methyl ester				1.838		
41.831	Octaethylene glycol			2.792		0.823	
41.936	Tetradecanoic acid	3.241					
41.998	1,3-Dioxan-5-ol						
42.193	Dimethyl phthalate						
42.331	Pentaethylene glycol			5.139			1.965
42.459	N-(2-Methoxyethyl)alanine					1.989	
42.578	Acetamide, 2-methoxy-N-(2-methoxyethyl)-			2.9			
42.593	2-[2-[2-(tert-Butyldimethylsilyloxy)ethoxy]ethoxy]ethanol				0.402		
42.678	Methoxyacetyl chloride			2.717			
42.8	Hexagol	1.455	1.074	1.475		2.321	
42.955	Octaethylene glycol monododecyl ether			1.631			0.907
43.021	3,6,9,12-Tetraoxatetradecan-1-ol						0.836
43.093	3,6,9-Trioxa-2-silaundecane, 2,2-dimethyl-			3.871			
43.197	Boronic acid, ethyl-, diethyl ester					1.191	
43.221	Cyclopropanetetradecanoic acid, 2-octyl-, methyl ester						
43.412	(R)-(-)-3-Methyl-2-butanol	1.471					
43.436	2-[2-[2-[2-(tert-Butyldimethylsilyloxy)ethoxy]ethoxy]ethoxy]ethanol			1.29			
43.802	Ethanol, 2-[2-(2-ethoxyethoxy)ethoxy]-					1.768	
43.864	Propane, 2-ethoxy-	1.171	0.681				
43.912	Methyl-2,3,5-tri-O-methyl-4-thio.alpha.d-arabinofuranoside					0.355	
44.298	Butane, 1,4-dimethoxy-					1.394	
44.631	2-[2-[2-[2-[2-[2-(2-Hydroxyethoxy)ethoxy]ethoxy]ethoxy]ethoxy]ethoxy]ethanol			1.524	0.467	1.137	
44.659	Propane, 1-methoxy-2-methyl-		0.505				3.081
44.7	Benzofuran, 2,3-dihydro-	1.214					
45.088	Diisopropyl ether					1.35	1.209

45.459	DL-Glyceraldehyde, dimethyl ether				0.268		1.432
45.464	13,13-Dimethyl-3,6,9-trioxa-13-silatetradecan-1-ol					0.392	
45.583	2-Propanol, 1-bromo-					0.283	
45.712	Ether, sec-butyl isopropyl			0.21			
45.774	2,4-Monoethylidene-l-xylitol		0.496				1.588
45.821	Ethanol, 2,2'-[oxybis(2,1-ethanediyloxy)]bis-	0.591					
45.897	11,13-Dihydroxy-tetradec-5-enoic acid, methyl ester			0.189	0.236		
46.088	3-Hexene, 1-[1-ethoxyethoxy]-, (E)-				0.142		
